# Peak Cenozoic warmth enabled deep-sea sand deposition

**DOI:** 10.1038/s41598-022-27138-2

**Published:** 2023-02-08

**Authors:** Zachary F. M. Burton, Tim McHargue, Christopher H. Kremer, Roger B. Bloch, Jared T. Gooley, Chayawan Jaikla, Jake Harrington, Stephan A. Graham

**Affiliations:** 1 Department of Geological Sciences, Stanford University, Stanford, CA 94305 USA; 2 Department of Earth, Environmental and Planetary Sciences, Brown University, Providence, RI 02912 USA; 3 U.S. Geological Survey, Alaska Science Center, Anchorage, AK 99508 USA

**Keywords:** Geology, Sedimentology

## Abstract

The early Eocene (~ 56–48 million years ago) was marked by peak Cenozoic warmth and sea levels, high CO_2_, and largely ice-free conditions. This time has been described as a period of increased continental erosion and silicate weathering. However, these conclusions are based largely on geochemical investigation of marine mudstones and carbonates or study of intermontane Laramide basin settings. Here, we evaluate the marine coarse siliciclastic response to early Paleogene hothouse climatic and oceanographic conditions. We compile an inventory of documented sand-rich (turbidite) deep-marine depositional systems, recording 59 instances of early Eocene turbidite systems along nearly all continental margins despite globally-elevated sea levels. Sand-rich systems were widespread on active margins (42 instances), but also on passive margins (17 instances). Along passive margins, 13 of 17 early Eocene systems are associated with known Eocene-age fluvial systems, consistent with a fluvial clastic response to Paleogene warming. We suggest that deep-marine sedimentary basins preserve clastic records of early Eocene climatic extremes. We also suggest that in addition to control by eustasy and tectonism, climate-driven increases in sediment supply (e.g., drainage integration, global rainfall, denudation) may significantly contribute to the global distribution and volume of coarse-grained deep-marine deposition despite high sea level.

## Introduction

Deep-marine sand-rich deposition may be promoted by sea-level fall (reduced accommodation) and by high sediment supply regardless of sea level (overwhelmed accommodation). However, neither the relative influence of sea level versus sediment supply nor the impact of climate on deep-sea siliciclastic sedimentation has been explored in the ancient record at a global scale. To investigate the potential for sediment supply to exceed accommodation at a global scale regardless of sea level and to investigate climatic influence on this sediment supply, we compile an inventory of documented sand-rich deep-sea systems active during the warm early Paleogene (during the highest sea levels of the Cenozoic). Our work likewise serves as an investigation of the deep-marine clastic sedimentary basin response to inferred increases in continental erosion and silicate weathering during the early Eocene^1–6^.

### The paradigm of deep-water siliciclastic deposition

Sequence stratigraphic models of coarse-grained siliciclastic sedimentation hold that sediment volume must exceed shelf accommodation for shoreline progradation and voluminous deep-sea deposition to occur^7,8^. Falling relative sea level and a resultant decrease in accommodation is the prevailing paradigm for delivering coarse clastic detritus to deep-water passive margin settings^9–11^. Nevertheless, coarse-grained deep-marine deposition during high sea level may occur at a local level (1) along narrow shelves of tectonically active margins^12–15^; (2) if shelf-penetrating canyons intersect the shoreline^12–14,16^; (3) during episodes of exceptionally voluminous sediment supply^15,17,18^; and (4) via other factors such as turbidite initiation by shelf undercurrents, longshore drift, orbital forcing, subglacial meltwater, and monsoons^13,19,20^. Despite such exceptions, and some debate about the general applicability of sequence stratigraphy^21^, the prevailing paradigm predicts minimal development of sand-rich deep-water systems along passive margins during high sea level. To test this hypothesis, we look to one of the most notable periods of elevated eustatic sea level of the Cenozoic: the warm early Eocene interval^22,23^.

### The early Eocene hothouse

The early Eocene (~ 56–48 Ma) was the warmest extended climate interval of the past 65 million years^24,25^. It was characterized by largely ice-free conditions, eustatic sea level at least 70–100 m above present, dampened, significantly lower-amplitude fluctuations in sea level (e.g., versus Quaternary glacial-interglacial fluctuations)^22,23^, subdued equator-to-pole temperature gradients^26,27^, and modifications to ocean^28^ and atmospheric circulation and hydroclimate^29,30^. If falling sea level is a prerequisite, the early Eocene would have been an especially unfavorable time for deep-sea sand-rich turbidite deposition on passive margins.

However, it has been proposed that this period was also characterized by high global-mean precipitation (a dominant control on marine sediment supply) and by more intense precipitation events than today^30–32^. These precipitation changes, along with elevated CO_2_ levels, contributed to increased chemical weathering^4,33,34^ and physical weathering and denudation^2,4,5^.

If the warm climate and intense precipitation of the early Eocene allowed sediment supply to outpace high accommodation, then sand-rich turbidites should be prevalent in the early Eocene depositional record, even on passive margins. Accordingly, our compilation reveals abundant examples of active margin but also passive margin sand-rich early Eocene (as well as Paleocene and middle Eocene) deep-sea systems. We present a conceptual model linking global climate and passive margin sand-rich turbidite systems and suggest tectonism and climatic conditions such as intense precipitation and integrated drainages provide the means to overwhelm eustasy and result in abundant sand-rich deep-marine deposition despite exceptionally high sea level.

### Abundant early Paleogene turbidite systems

**Figure 1:**
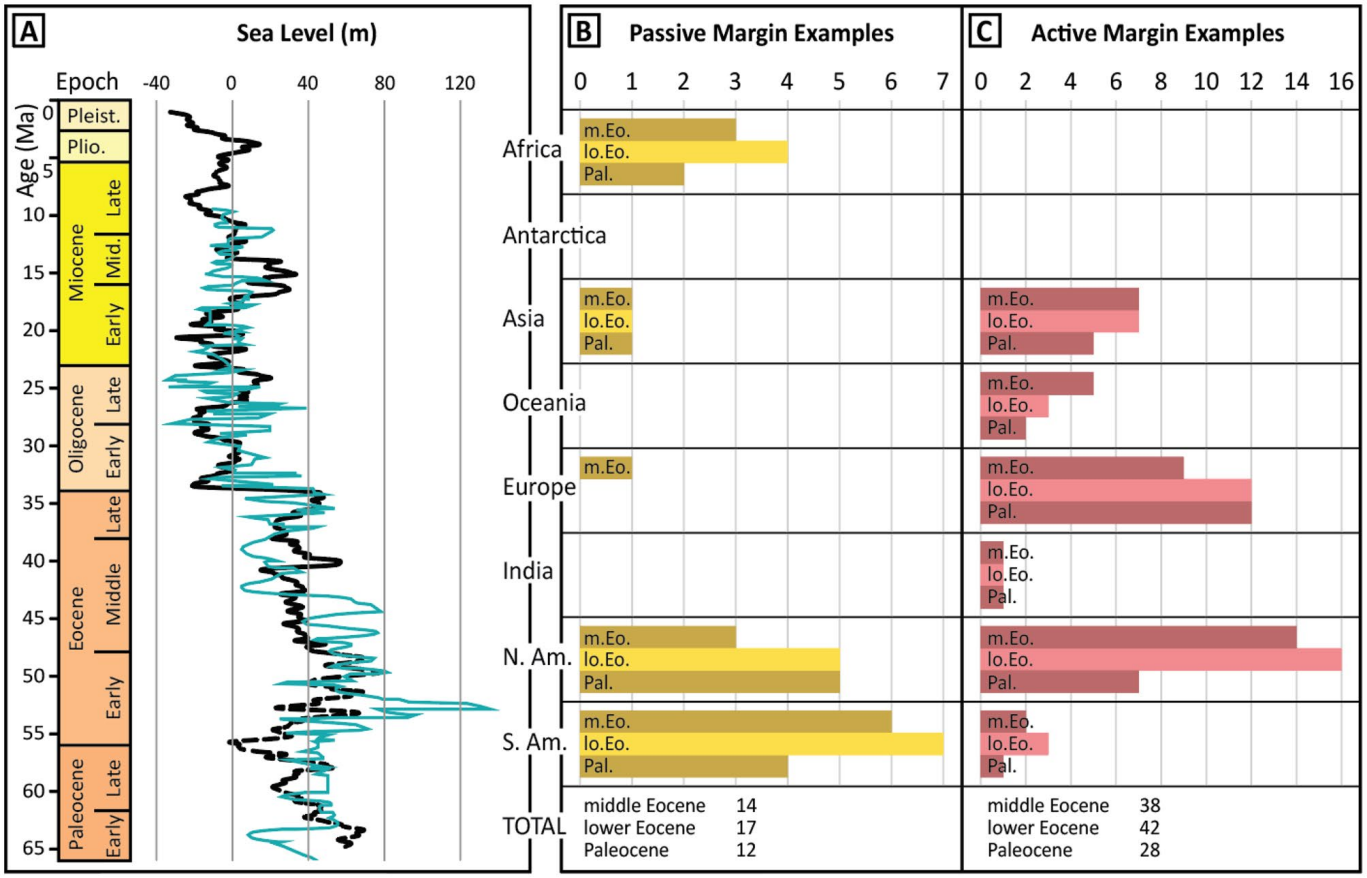
(**A**) Cenozoic sea-level curves of Miller et al.^22^ in cyan and Miller et al.^23^ in black. Bar charts with counts of (**B**) passive and (**C**) active margin sand-rich deposits for the Paleocene (Pal.), lower Eocene (lo. Eo.), and middle Eocene (m. Eo.) by continental margin. N. Am.—North America, S. Am.—South America. Location names and references are available in Supplementary Information.

**Figure 2:**
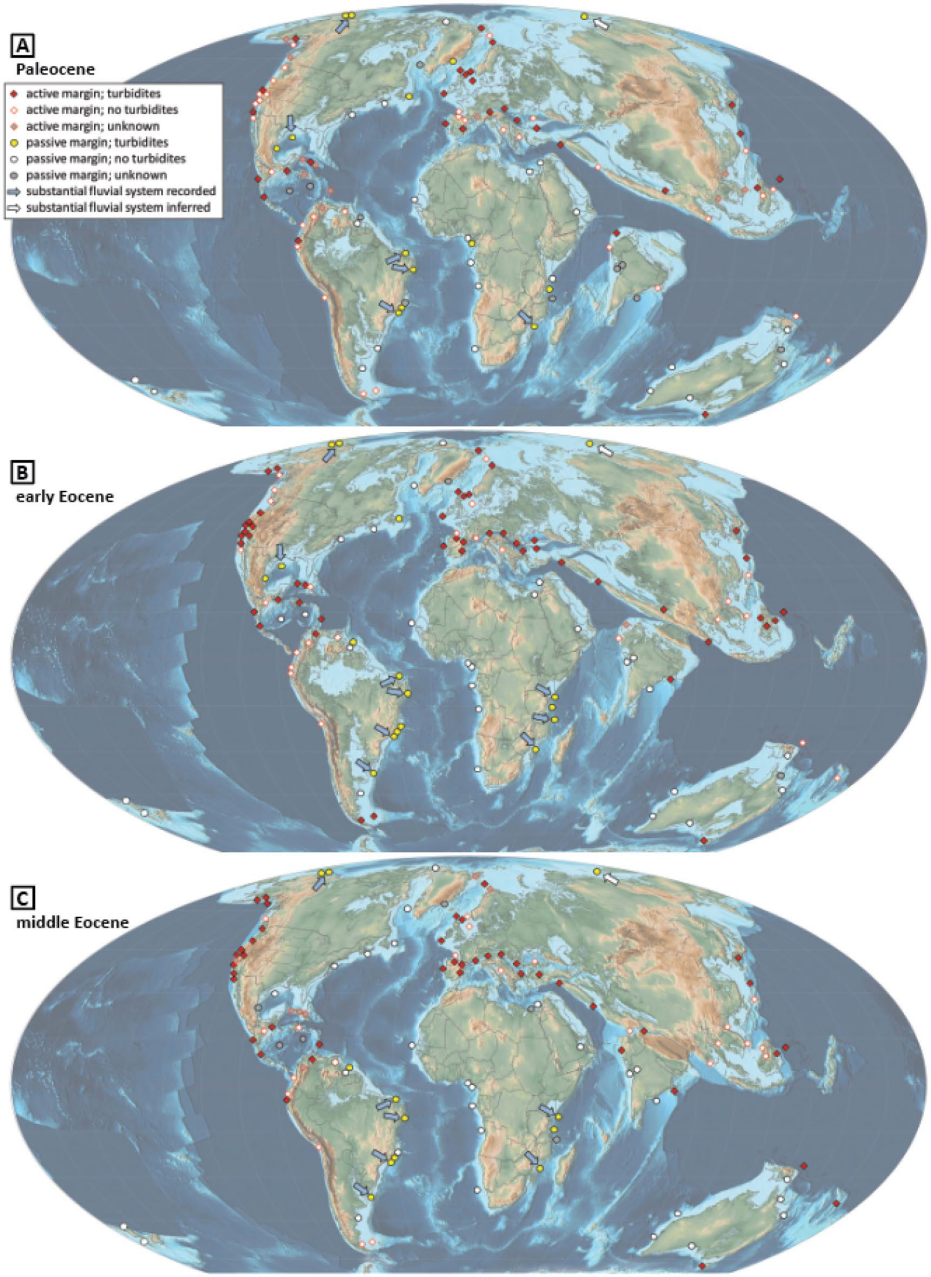
(**A**) Paleocene, (**B**) early Eocene, and (**C**) middle Eocene locations studied, including locations with and without recorded sand-rich deep-sea systems. Paleomaps are modified from the PALEOMAP Project of C. R. Scotese^35^. Location names and references are available in Supplementary Information.

Our survey identified 59 locations where early Eocene deep-sea sedimentation included sand-rich turbidite systems despite prevailing high sea levels (Figs. 1 and 2B). These locations span all continents except Antarctica, for which data was lacking. Most systems are in active margin settings (42 examples), but 17 examples of sand-rich passive margin turbidite systems were also identified (Figs. 1 and 2B). There are slightly more early Eocene examples than Paleocene (28 on active margins, 12 on passive margins; Figs. 1 and 2A) or middle Eocene (38 on active margins, 14 on passive margins; Figs. 1 and 2C) examples, although the proportion of passive versus active margin turbidite systems is similar. Location names and references for surveyed deep-marine depositional systems are available in Supplementary Information.

Sand-rich lower Eocene deep-sea deposits are found in active margin settings in Asia, Europe, India, and North and South America (Figs. 1C and 2B). In Europe and Asia, Alpine-Zagros-Himalayan collisional tectonism resulted in widespread and abundant foreland basin turbidite deposition. Similarly, the Pyrenean orogeny of Europe, Andean orogeny of South America, and Cordilleran orogenesis of North America contributed to turbidite deposition. Deposition also occurred during periods of rifting (e.g., southeast Borneo; Tasmanian shelf; west Ireland) and in borderlands settings (e.g., southern California; Ireland’s Porcupine Basin).

In both active and passive margin settings, deep-marine siliciclastic systems are often associated with noteworthy early Eocene fluvial systems. Of the 59 locations with recorded early Eocene turbidite systems, 19 (~ 32%) locations are explicitly documented as being associated with substantial fluvial systems. Of these examples, six are from active margin settings. Notably, 13 of 17 (~ 76%) locations in passive margin settings are associated with integrated ancestral fluvial systems (i.e., drainage networks interconnected by through-going river systems). Localized tectonism can also contribute sediment through these integrated drainages in passive margin settings. For five of the 17 passive margin locations (Tanzania Coastal Basin, Gulf of Mexico, and three Brazilian basins; Supplementary Information Table S1), authors cited possible influence of local tectonism (e.g., hinterland uplift, salt movement).

### Mechanisms of sand-rich turbidite deposition during eustatic highs

Widespread sand-rich early Eocene turbidite systems along both active and passive margins indicate that the falling sea-level paradigm^9–11^ need not be invoked for sediment supply to overwhelm shelf accommodation.

Alongside widespread evidence for active margin deposition during a eustatic high (e.g., as in Quaternary-age examples from California and Chile^12,14^ and as previously discussed from a process standpoint^36^), widespread distribution of early Paleogene turbidite systems suggests that in passive margin settings during eustatic highs, an integrated drainage system may be a prerequisite for substantial sand-rich deep-sea deposition^37^. Activity of such drainages may have been magnified by increasingly episodic, intense precipitation events in the early Eocene^30–32^, while sediment transport along drainages may have been magnified by intensified weathering and erosion^2–5,33,34^, both of which would act to increase sediment supply, and both of which could in turn drive further integration of drainages^38–41^. Increased sediment supply as a means to overwhelm shelf accommodation even during a highstand is consistent with work on high-supply and supply-dominated systems, whereby significant deep-marine turbidite deposition occurs despite eustatic highs (e.g., modern river estimates^17^; Maastrichtian shelf of Wyoming^18^; modeling work^15^), and may be consistent with work suggesting sediment supply as the dominant control—beyond frequency and amplitude of sea-level fluctuations—on deep-sea fan size^42,43^. Our findings also seem compatible with recent work documenting narrower shelves in greenhouse versus icehouse climates, wherein authors suggest that greenhouse deltas perched near the clinoform break of slope would promote significant all-stand sediment bypass into deep water^44^.

In consideration of the controls on sediment delivery to active and passive margins broadly speaking, it should be kept in mind that basin area and relief (i.e., existing or preexisting tectonic factors), alongside lithology and ice erosion, have been found to account for a majority of the variability in sediment load to modern global rivers, with climatic factors accounting for a lesser proportion of the variability in sediment load^36,45^. Thus, potential control by extreme early Eocene climate over sediment supply and deep-sea deposition should here be thought of as climatic controls overprinted on the tectonic factors and lithologies of the localities examined herein.

Furthermore, alongside influence of tectonism on sea level on active margins (e.g., uplift-driven falls in relative sea level) and the potential influence of localized tectonics on relative sea level on some passive margins, it should be noted that additional controls on local or global sea level fluctuations within the early Eocene (and adjacent Paleocene and middle Eocene) could exert influence on the development of the sand-rich depositional systems recorded here. For instance, although sea-level fluctuations are inferred to have been dampened and of significantly lower amplitude during the early Eocene, fluctuations are still inferred to have been present^22,23^. As such, further work on the scale and potential influence of early Eocene sea-level fluctuation is warranted, as is work characterizing local Eocene sea-level histories (e.g., potential evidence for local sea-level fall) at individual localities catalogued within this work.

### Conceptual model linking global climate and sand-rich turbidite systems

**Figure 3:**
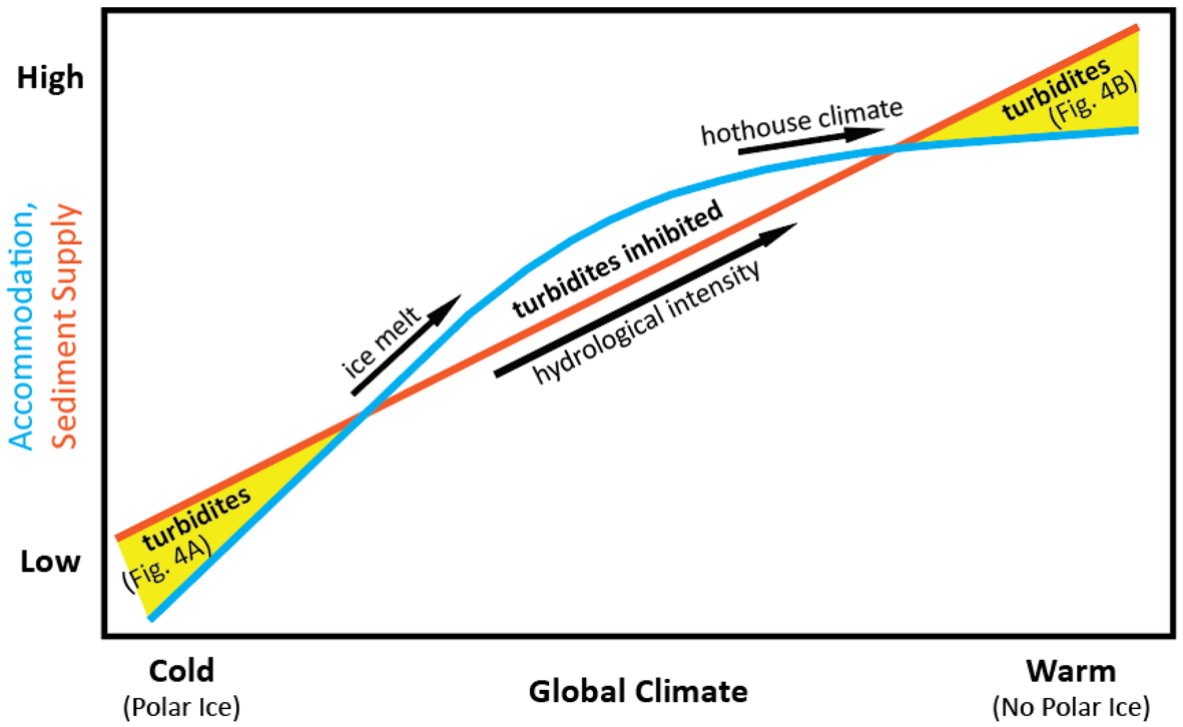
Conceptual model of passive margin sandy turbidite deposition related to climate controls on sediment supply (orange line) and accommodation (blue curve). Sediment supply increases with global temperature via its role in intensifying precipitation. Accommodation increases rapidly with initial warming as ice melts and sea level rises. Later warming (after ice melt) has a much weaker effect on sea level and accommodation. Sand-rich deposition (yellow areas) happens when sediment supply exceeds accommodation for a given climate state.

**Figure 4:**
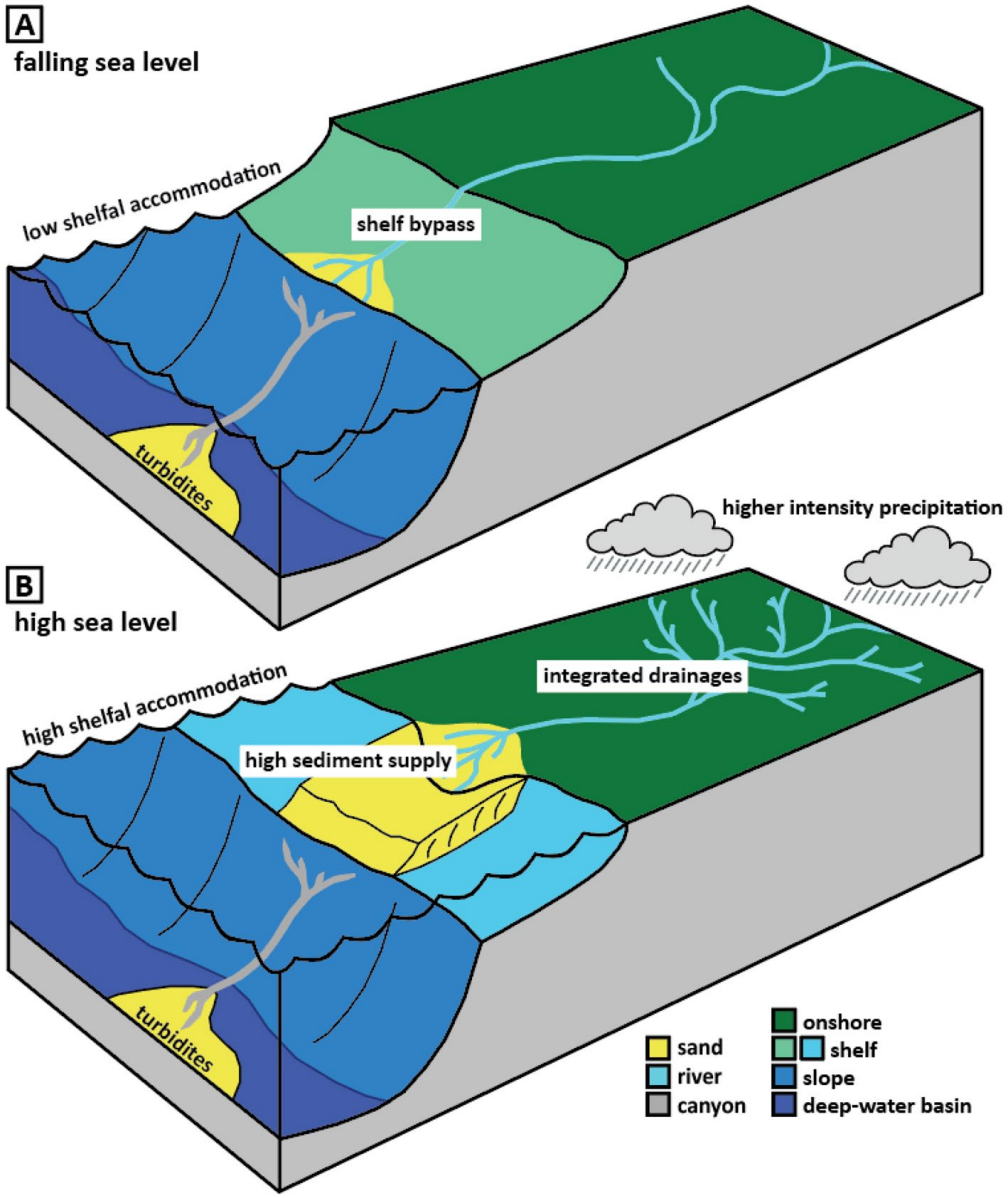
Schematic illustration of sand-rich passive margin deep-sea deposition under (**A**) falling sea level, where a drop in shelfal accommodation drives deep-water turbidite deposition, and (**B**) high sea level, where climatically enhanced sediment supply overwhelms high accommodation.

Based on our results, we present a conceptual model where sandy turbidite occurrence is related to climatic controls on sediment supply and accommodation (Figs. 3 and 4). Our model is compatible both with the falling sea level paradigm for deposition (Fig. 4A) and with deposition during eustatic highs (Fig. 4B). For simplicity, Fig. 3 shows only relative changes on a global scale (i.e., our model does not attempt to quantify the magnitude of change, nor does it consider influence of local variables such as local uplift and deformation on otherwise tectonically quiescent margins). We assume that sediment supply is a linear function of precipitation intensity (a conceptually useful but oversimplified assumption^46^), accommodation is a function of sea level, and both precipitation intensity and sea level can be linked directly to global climate. We justify these assumptions below.

As the planet warms, precipitation intensifies and becomes increasingly episodic^31^, resulting in a direct relationship between temperature and sediment supply. Starting from a cold climate, warming leads to a relatively rapid increase in eustatic sea level (thus, in accommodation) due to ice sheet melt. However, after the loss of large continental glaciers, additional warming leads to smaller thermal-expansion-driven increases in sea level^47^. Turbidite deposition occurs in our model when, for a given climate state, sediment supply (orange line; Fig. 3) exceeds accommodation (blue curve; Fig. 3).

Numerous factors influence the slope and intercept of the supply and accommodation lines without violating the basic conceptual framework. For example, intra-continental orogenesis, greater drainage size (capturing more sediment), or drainages with more active canyon incision can increase the y-intercept of the sediment supply line. The slope of the supply line may decrease in very hot climates because precipitation is theorized to decline with extreme warming^48^. Meanwhile, the accommodation curve is sensitive to factors that modify relationships among sea level, ice sheet, and climate. If continental water storage can drive high-amplitude sea-level variability without ice sheets^47^, the slope of the accommodation curve would be modified at warmer temperatures. These climatic and tectonic factors imply that the shape and relative locations of the supply and accommodation curves in our model vary over space and time. Therefore, our framework may be adapted to account for future constraints on climate, erosion, and accommodation dynamics when inferring turbidite occurrence.

### Future research directions

Our investigation invites research related to both deep-sea turbidite deposition during eustatic highs and our model linking climate and sand-rich systems (for instance, can we indeed use passive margin turbidite occurrence to predict the nature of sediment supply and the potential for, or necessity of, drainage integration?). Our results and model invite compilations for other hothouse and warm periods, as in the Cretaceous^49,50^ or Paleozoic^51,52^, to further query the potential for widespread sandy, deep-marine systems during eustatic highs, as well as compilations for periods of falling or low sea level (e.g., cooling or cold climates) to further test our conceptual model. Furthermore, in light of anthropogenic climate change, our work invites investigation into the ramifications of present-day climate warming and warming-driven increases in sediment supply^41,53,54^ on deep-sea sedimentary systems.

### Concluding remarks on sea level versus sediment supply

Our inventory of Paleogene deep-sea sand-rich systems joins other studies, including global tabulations of modern and geologically recent systems, examples of specific ancient systems, and modeling studies, that have challenged the notion that sea-level change (eustasy) is the prevailing control on deep-marine coarse siliciclastic deposition. Using the warmest interval of the Cenozoic—the early Paleogene, and especially early Eocene—as a “high sea level endmember,” we show in deep time and at global scale that sediment supply can exceed accommodation on both passive and active margins. Our data show that despite extremely high sea level, sand-rich systems occur on passive margins of all continents (except Antarctica, for which we have no data) during the hot early Eocene, as well as the warm Paleocene and middle Eocene. Thus, in considering the relative influence of sea level versus sediment supply, we conclude that sediment supply is at least as important as global eustasy during high sea level. This is consistent with high-supply or supply-dominated systems documented by previous authors^15,17,18,55^. We suggest that tectonics, large integrated fluvial drainages on a local to regional scale, and intensification of the hydrological cycle on a global scale can combine to deliver sufficient coarse-grained sediment to passive margins to overwhelm sea level-driven high shelf accommodation and result in widespread sand-rich deep-marine deposition. Eustasy need not be the dominant control on sand-rich deep-sea turbidite deposition on a global scale—and factors such as climatic forcing of sediment supply warrant more consideration than previously granted.

## Methods

We reviewed existing literature to compile a global inventory of published examples of marine (primarily deep marine) sedimentary deposits of Paleocene, early Eocene, and middle Eocene age. We report records of deposition at 112 global sites (including 9 sites from Africa, 0 sites from Antarctica, 15 sites from Asia, 14 sites in Australia/Oceania, 15 sites in Europe, 6 sites in India, 34 sites in North and Central America, and 19 sites in South America; see Supplementary Information for full location names and references), though it should be noted that we surveyed many more sites that we have here excluded because they contained no record of Paleocene, lower Eocene, and/or middle Eocene deep-water deposits (e.g., the margins of Antarctica). Furthermore, a number of the 112 sites we did survey themselves represent numerous locations, basins, and/or geographic regions (e.g., the rift basins of eastern India are grouped together).

Our database relies on published examples of marine turbidites and deep-water sediments of Paleocene through Eocene age. These examples almost entirely consist of onshore outcrop studies and offshore drilling studies, in which sedimentological/lithological assessment (e.g., identification of turbidites) has been performed based on direct access to Paleocene, lower Eocene, and/or middle Eocene rock, although in rare instances we include examples of inferred turbidite sediment occurrence based on published analysis of seismic data (e.g., poorly explored regions such as the East Siberian Shelf).

Because our study is in essence a compilation of previously published data and interpretations, it inherently relies on the validity and accuracy of this previously published work. We rely on the interpretations of previous authors in aspects including the interpreted age of sediment (e.g., geochronology, biostratigraphy work), the interpreted sedimentology and lithology of sediment, the interpreted depositional environment of sediment, and some aspects of associated local and regional tectonic and climatic associations.

Our study is also impacted by limitations in the availability and comprehensiveness of published examples of deep marine sedimentation, including examples of turbidites. An assumption inherent to compiling a global database of such examples is that existing published examples are representative of the true global distribution of examples of such marine sediments. However, our record, and the published record in general, is most certainly incomplete, and most certainly underrepresents the total number of turbidite and deep-water sediment occurrences of any given age. Furthermore, we acknowledge that we likely missed examples of turbidites of this age published in non-English language sources. Thus, our compilation provides a lower bound to the total number of turbidite deposits from the Paleocene, early Eocene, and middle Eocene.

A further limitation to our study is that many regions of the world, including remote onshore terrains and difficult-to-access offshore basins, are currently poorly explored (e.g., much of the Arctic). Some of these regions may have further examples of Paleocene- through middle Eocene-age turbidite deposits. Similarly, there may be turbidites of this age that have been encountered during drilling and exploration efforts by oil and gas companies across the world, but are not reported in the published literature.

Poor preservation of sedimentary deposits from the Paleogene may pose a further limitation to the number of recorded Paleogene turbidites. For instance, some Paleocene- and Eocene-age turbidites may have been subducted along active margins and thus lost to the rock record. Similarly, sediment of this age may have been eroded subsequent to deposition. Any lack of preservation, as with a lack of exploration, would potentially yield underestimation of the total number of Paleocene, lower Eocene, and middle Eocene turbidites.

## Supplementary Information

Supplementary Information.

## Data Availability

All raw data (references) used for this manuscript are included within the Supplementary Information file, ensuring the replicability of this study.
