# Supplementary material for: Peak Cenozoic warmth enabled deep-sea sand deposition
Source: Sci Rep. 2023 Feb 8;13:1276. doi: 10.1038/s41598-022-27138-2 (PMC9908870; doi:10.1038/s41598-022-27138-2)
Supplement: Supplementary file 1 — Supplementary Information. [file 41598_2022_27138_MOESM1_ESM.pdf]

SUPPLEMENTARY INFORMATION

Peak Cenozoic warmth enabled deep-sea sand deposition

Zachary F.M. Burton[1,*], Tim McHargue[1], Christopher H. Kremer[2], Roger B. Bloch[1], Jared T. Gooley[3], Chayawan Jaikla[1], Jake Harrington[1], Stephan A. Graham[1]

[1] Department of Geological Sciences, Stanford University, Stanford, CA 94305, USA

[2] Department of Earth, Environmental & Planetary Sciences, Brown University, Providence, RI 02912, USA

[3] Alaska Science Center, U.S. Geological Survey, Anchorage, AK 99508, USA

* Corresponding author. E-mail address: zburton@stanford.edu (Z.F.M. Burton)

SUPPLEMENTARY INFORMATION CONTENTS

Table S1: Basic metadata for documented early Eocene sand-rich systems page 3

Table S2: Basic metadata for sites lacking early Eocene sand-rich systems page 6

References: Table S1 page 9

References: Table S2 page 26

Table S1: Basic metadata for the 59 documented early Eocene sand-rich systems.

	Location number	Location name	Reference(s)
**Africa**	1	Zambezi Delta (Mozambique Basin)	Salman and Abdula, 1995; Walford et al., 2005; Said et al., 2015; Castelino et al., 2017
	2	Northern Mozambique (Rovuma Basin)	Palermo et al., 2014; Palermo et al., 2015
	3	Tanzania Coastal Basin	Sansom, 2018
	4	Southeast Kenya (Lamu Basin)	Nyagah, 1995; Zongying et al., 2013
**Asia**	5	Northwest Turkey (Thrace Basin)	Siyako and Huvaz, 2007
	6	Central Turkey basins (Haymana, Tuzgölü, etc.)	Görür et al., 1984; Görür et al., 1998
	7	Zagros (southeastern Turkey, northern Syria and Iraq, western and southern Iran)	Koop and Stoneley, 1982; Alavi, 2004
	8	Central Palawan Island	Suzuki et al., 2000
	9	Southern Palawan Island	Steuer et al., 2013; Aurelio et al., 2014
	10	Shimanto Supergroup (outboard of East China Sea)	Taira et al., 1982
	11	Shimanto Supergroup (Okinawa Island)	Ujiie, 2002
	12	Northwestern East Siberian Shelf (bordering Makarov Basin)	Sekretov, 2001
**Europe**	13	Spitsbergen/Svalbard (Central Basin)	Steel et al., 1985; Grundvåg et al., 2014
	14	Southwestern Barents Sea (Tromsø and Hammerfest basins)	Knutsen and Vorren, 1991; Knutsen et al., 1992
	15	Northern and central North Sea (between Shetland Platform and Norwegian Shield)	Heritier et al., 1979; Morton, 1982; den Hartog Jager et al., 1993
	16	North Sea Viking Graben (Frigg fan)	Heritier et al., 1979; McGovney and Radovich, 1985
	17	Faroe-Shetland Basin	Lamers and Carmichael, 1999; Sørensen, 2003; Davies et al., 2004
	18	Western Ireland (Porcupine Basin)	Croker and Klemperer, 1989; Moore and Shannon, 1992; Shannon, 1992
	19	Southern Pyrenean foreland basin	Mutti, 1977; Mutti, 1983; Labaume et al., 1985

	20	Pyrenees (Ainsa, Pamplona, Basque basins, coast of Bay of Biscay)	Gawenda et al., 1999; Payros et al., 2006; Pickering and Bayliss, 2009; Payros and Martínez-Braceras, 2014
	21	Offshore central west Iberia (Peniche Basin)	Alves et al., 2006
	22	Southern Iberian margin (southern Spain)	De Ruig et al., 1991
	23	Western, central, and eastern Alps	Trümpy, 1960; Hesse, 1975; Faupl and Wagreich, 2000
	24	Carpathians (e.g., Poland's Ciezkowice Sandstone)	Dziadzio et al., 2006
**India**	25	Bengal submarine fan	Alam et al., 2003; Curray et al., 2003; Curray, 2014
**Oceania**	26	Northwest Borneo	Moss, 1998
	27	Southeast Borneo (Kutai Basin)	Camp et al., 2009
	28	West Tasmania shelf	Exon et al., 2004
**North America**	29	San Joaquin Basin	Nilsen et al., 1974; Dickinson et al., 1979; Graham and Berry, 1979, Reid, 1988
	30	Sacramento Basin	Redwine, 1972; Dumitru et al., 2013
	31	Borderlands basins (Gualala, Sierra Madre, Santa Ynez basins)	Nilsen and Clarke, 1975
	32	Borderlands (La Honda Basin, Santa Cruz Mountains)	Nilsen and Simoni, 1973; Nilsen, 1985
	33	Borderlands (N Santa Lucia Range)	Link and Nilsen, 1980
	34	Northern California Yager complex	Underwood, 1983; Dumitru et al., 2013
	35	Oregon Tyee Basin	Chan and Dott, 1983; Dickinson et al., 1988; Dumitru et al., 2013; Santra et al., 2013
	36	Northern Gulf of Alaska	Winkler, 1976; Plafker, 1987
	37	Northern Gulf of Alaska (Kodiak Island)	Bradley et al., 2003
	38	Southern Beaufort Sea (Beaufort-Mackenzie Basin)	Willumsen and Cote, 1982
	39	Beaufort Sea/Arctic Ocean (Southern Canada Basin)	Tailleur, 1970
	40	Western and southern Jeanne d'Arc Basin	Deptuck et al., 2003
	41	Eastern and northeastern Jeanne d'Arc Basin	Deptuck et al., 2003

	42	Gulf of Mexico	Winker, 1982; Galloway et al., 2000; Galloway et al., 2005; Galloway et al., 2011; Sharman et al., 2017; Zhang et al., 2018
	43	Western Cuba (Los Palacios Basin)	Brust et al., 2011; Villegas-Martín et al., 2014
	44	Central Cuba	Menéndez et al., 2011
	45	Southeastern Hispaniola (southeastern Peralta Belt)	Dolan et al., 1991
	46	Eeastern Barbados	Speed, 1981; Pudsey and Reading, 1982; Speed and Larue, 1982
	47	Southern Belize/Eastern Guatemala (Belize Basin)	Ramanathan and Garcia, 1991; Schafhauser et al., 2003
	48	Western Nicaragua (coastal and offshore Sandino forearc basin)	Swain, 1966; Kumpulainen, 1995; Ranero et al., 2000; Struss et al., 2007; Struss et al., 2008
	49	Eastern Costa Rica (Limón Basin)	Bowland, 1993; Brandes et al., 2007
**South America**	50	Venezuela (Maracaibo foreland basin)	Talukdar et al., 1986; Lugo and Mann, 1995; Escalona and Mann, 2006; Xie et al., 2010
	51	Offshore Guyana-Suriname Basin	Nemčok et al., 2016
	52	Brazil (Sergipe Basin)	Karner and Driscoll, 1999
	53	Brazil (Mundaú Basin)	Karner and Driscoll, 1999
	54	Brazil (Espírito Santo Basin)	Bruhn and Walker, 1997
	55	Brazil (Campos Basin)	Guardado et al., 1989; Mohriak et al., 1990
	56	Brazil (Northern and central Santos Basin)	Modica and Brush, 2004; Berton and Vesely, 2016
	57	Uruguay (Punta del Este and Pelotas basins)	Contreras et al., 2010; Soto et al., 2011
	58	Austral Basin	Olivero and Malumián, 2008; Torres Carbonell and Olivero, 2012
	59	Malvinas Basin	Olivero and Malumián, 2008; Torres Carbonell and Olivero, 2012

Table S2: Basic metadata for sites lacking early Eocene sand-rich systems.

	Location number	Location name	Reference(s)
**Africa**	60	Western African margin (Senegal Basin to Orange Basin)	Seranne and Abeigne, 1999; Brownfield and Charpentier, 2003; Monnier et al., 2014
	61	Niger delta fan	Damuth, 1994; Tuttle et al., 1999; Macgregor, 2012
	62	Equatorial Guinea	Macgregor, 2012; Thornton et al., 2013
	63	Western South Africa (Orange River)	Paton et al., 2010
	64	Eastern and central Sahara (Egypt, Libya, Chad, Niger, Algeria, Tunisia)	Swezey, 2009
**Asia**	65	Levantine Basin (offshore Turkey, Syria, Lebanon, Israel, Egypt)	Gardosh and Druckman, 2006
	66	Around the Arabian Plate	Alsharhan and Nairn, 1995; Brannan et al., 1997; Beavington-Penney et al., 2006; Garzanti et al., 2013
	67	Thailand	Polachan et al., 1991; Charusiri and Pum-Im, 2009
	68	Southern and central Vietnam (South China Sea Basin)	Lee and Watkins, 1998; Lee et al., 2001
	69	Northern Vietnam and southern China (South China Sea Basin)	Chen et al., 1993
	70	Taiwan (South China Sea Basin)	Lin et al., 2003; Huang et al., 2012
	71	Eastern China intracontinental basins, China eastern marginal seas basins (including East China Sea Basin, Yellow Sea basins), Sea of Japan	Ren et al., 2002
**Europe**	72	Southwestern Barents Sea (Sørvestsnaget Basin)	Ryseth et al., 2003; Henriksen et al., 2011; Safronova et al., 2014
	73	Southeastern Greenland (Kangerlussuaq Basin)	Larsen et al., 2006
	74	Offshore Bay of Biscay/French Atlantic coast	Bourillet et al., 2006
**India**	75	Afghanistan (northern Katawaz Basin), Khojak fan	Qayyum et al., 1997a, 2001
	76	Pakistan (Katawaz Basin) and Iran (Makran)	Qayyum et al., 1996, 1997b, 2001; Carter et al., 2010
	77	Western India (Kutch Basin)	Biswas, 1982

	78	Western India (Cambay Basin)	Biswas, 1982
	79	Eastern India rift basins	Sastri et al., 1973; Bastia, 2006
**Oceania**	80	Northeastern Papua New Guinea (Finisterre Range)	Abbott et al., 1994
	81	New Caledonia	Maurizot, 2011; Maurizot and Cluzel, 2014
	82	New Zealand (Rotorua, Raukumara regions)	Morris, 1987; Kamp et al., 2015
	83	New Zealand (Kaikoura area)	Morris, 1987; Kamp et al., 2015
	84	New Zealand (Kaikoura, Christchurch regions)	Morris, 1987; Kamp et al., 2015
	85	Southern Australia (Eucla Basin)	Hou et al., 2006
	86	Western Australia	Quilty, 1977
	87	Northwest shelf, Australia	Butcher, 1990; Baillie et al., 1994
	88	Northeastern Australia (Gulf of Papua)	Davies et al., 1989; Feary et al., 1991
	89	Northeastern Australia (Queensland Plateau)	Davies et al., 1989
	90	Northeastern Australia (Capricorn Basin)	Davies et al., 1989
**North America**	91	Washington State	Armentrout and Suek, 1985; Snavely, 1987; Snavely and Wells, 1996
	92	Vancouver/Queen Charlotte islands region	Tiffin et al., 1972; Rohr and Dietrich, 1992; Bustin, 1995
	93	Southern and central Gulf of Alaska	Hamilton, 1967; Stevenson and Embley, 1987
	94	Bering Sea (Aleutian Basin, northern basins, etc.)	Scholl et al., 1975; Fisher et al., 1982
	95	Arctic Canada (Sverdrup Basin)	Balkwill, 1978
	96	East Greenland rift margin	Eldrett et al., 2004
	97	Western Greenland/eastern Canadian shelf (Labrador Sea)	Beh, 1975; Gradstein and Srivastava, 1980; McWhae et al., 1980; Schenk, 2011
	98	Scotian Basin and Shelf	McIver, 1972; Grant et al., 1986
	99	Baltimore Canyon Trough/Hatteras Basin	Poag and Sevon, 1989
	100	North-central Cuba foredeep basin	Iturralde-Vinent et al., 2008
	101	Venezuela Basin (Caribbean Sea)	Ewing et al., 1967; Bader et al., 1970; Edgar et al., 1973; Bowland, 1993
	102	Central Colombia Basin (Caribbean Sea)	Ewing et al., 1967; Bader et al., 1970; Edgar et al., 1973; Bowland, 1994

	103	Northeastern Honduras/northern Nicaragua (Mosquita Basin)	Mills and Hugh, 1974; Mills and Barton, 1996
**South America**	104	Eastern Venezuela Basin	Di Croce, 1996
	105	Onshore Guyana-Suriname Basin	Toelsie and Goerdajal, 2013
	106	Offshore central Argentina basins	Bushnell et al., 2000; Franke et al., 2006; Gruetzner et al., 2012
	107	Peru-Chile Trench	Scholl et al., 1968; Scholl et al., 1970
	108	Peru continental margin (ten Mesozoic/Cenozoic basins)	Travis et al., 1976; Dunbar et al., 1990
	109	Peru (Talara Basin)	Travis et al., 1976; Marsaglia and Carozzi, 1990; Fildani et al., 2008
	110	Ecuador forearc regions	Nygren, 1950; Jaillard et al., 1995; Jaillard et al., 2000; Alava and Jaillard, 2005; Marcaillou and Collot, 2008
	111	Northern Ecuador-southern Panama (Pacific Coastal Basin)	Bueno Salazar and Govea, 1974; Bueno Salazar, 1989
	112	Middle Magdalena Valley, Eastern Cordillera, and eastern Llanos Basin	Cooper et al., 1995

References: Table S1

Alam, Mahmood, M. Mustafa Alam, Joseph R. Curray, M. Lutfar Rahman Chowdhury, and M. Royhan Gani. "An overview of the sedimentary geology of the Bengal Basin in relation to the regional tectonic framework and basin-fill history." *Sedimentary Geology* 155, no. 3-4 (2003): 179-208.

Alavi, Mehdi. "Regional stratigraphy of the Zagros fold-thrust belt of Iran and its proforeland evolution." *American Journal of Science* 304, no. 1 (2004): 1-20.

Alves, Tiago M., Carlos Moita, Frode Sandnes, Tiago Cunha, José H. Monteiro, and Luis M. Pinheiro. "Mesozoic–Cenozoic evolution of North Atlantic continental-slope basins: The Peniche basin, western Iberian margin." *AAPG Bulletin* 90, no. 1 (2006): 31-60.

Aurelio, Mario A., Monina T. Forbes, Kristine Joy L. Taguibao, Raymundo B. Savella, Jaime A. Bacud, Dieter Franke, Manuel Pubellier et al. "Middle to Late Cenozoic tectonic events in south and central Palawan (Philippines) and their implications to the evolution of the south-eastern margin of South China Sea: Evidence from onshore structural and offshore seismic data." *Marine and Petroleum Geology* 58 (2014): 658-673.

Berton, Fábio, and Fernando F. Vesely. "Stratigraphic evolution of Eocene clinoforms from northern Santos Basin, offshore Brazil: Evaluating controlling factors on shelf-margin growth and deep-water sedimentation." *Marine and Petroleum Geology* 78 (2016): 356-372.

Bowland, Christopher L. "Depositional history of the western Colombian Basin, Caribbean Sea, revealed by seismic stratigraphy." *GSA Bulletin* 105, no. 10 (1993): 1321-1345.

Bradley, Dwight, Tim Kusky, Peter Haeussler, Rich Goldfarb, Marti Miller, Julie Dumoulin, Steven W. Nelson, and Sue Karl. "Geologic signature of early Tertiary ridge subduction in Alaska." (2003) in V. B. Sisson, S. M. Roeske, and T. L. Pavlis, eds., Geology of a Transpressional Orogen Developed During Ridge–Trench Interaction Along the North Pacific Margin: GSA Special Papers 371, pp. 19-49.

Brandes, Christian, Allan Astorga, Stefan Back, Ralf Littke, and Jutta Winsemann. "Deformation style and basin-fill architecture of the offshore Limón back-arc basin (Costa Rica)." *Marine and Petroleum Geology* 24, no. 5 (2007): 277-287.

Bruhn, Carlos H. L., and Roger G. Walker. "Internal architecture and sedimentary evolution of coarse-grained, turbidite channel-levee complexes, Early Eocene Regencia Canyon, Espirito Santo Basin, Brazil." *Sedimentology* 44, no. 1 (1997): 17-46.

Brust, Juliane, Heiko Hüneke, Martin Meschede, and Max Sommer. "Facies and provenance of basin-margin deposits in the Los Palacios Basin (Capdevila Formation, Cuba)." *Facies* 57, no. 1 (2011): 73-92.

Camp, Wayne K., Elly E. Guritno, Deden Drajat, and Moyra E. J. Wilson. "Middle-lower Eocene turbidites: a new deepwater play concept, Kutei Basin, East Kalimantan, Indonesia." (2009) Proceedings of the Indonesian Petroleum Association 33rd Annual Convention 1–19 p.

Castelino, Jude A., Christian Reichert, and Wilfried Jokat. "Response of Cenozoic turbidite system to tectonic activity and sea-level change off the Zambezi Delta." *Marine Geophysical Research* 38 (2017): 209-226.

Chan, M. A., and R. H. Dott Jr. "Shelf and Deep-Sea Sedimentation in Eocene Forearc Basin, Western Oregon–Fan or Non-Fan?" *AAPG Bulletin* 67, no. 11 (1983): 2100-2116.

Contreras, Jorham, Rainer Zühlke, Scott Bowman, and Thilo Bechstädt. "Seismic stratigraphy and subsidence analysis of the southern Brazilian margin (Campos, Santos and Pelotas basins)." *Marine and Petroleum Geology* 27, no. 9 (2010): 1952-1980.

Croker, Peter F., and Simon L. Klemperer. "Structure and Stratigraphy of the Porcupine Basin: Relationships to Deep Crustal Structure and the Opening of the North Atlantic: Chapter 29: European-African Margins." (1989) in A. J. Tankard and H. R. Balkwill, eds., Extensional Tectonics and Stratigraphy of the North Atlantic Margins: AAPG Memoir 46, pp. 445-459.

Curray, Joseph R., Frans J. Emmel, and David G. Moore. "The Bengal Fan: morphology, geometry, stratigraphy, history and processes." *Marine and Petroleum Geology* 19.10 (2003): 1191-1223.

Curray, Joseph R. "The Bengal depositional system: from rift to orogeny." *Marine Geology* 352 (2014): 59-69.

Davies, Richard, Ian Cloke, Joe Cartwright, Andrew Robinson, and Charles Ferrero. "Post-breakup compression of a passive margin and its impact on hydrocarbon prospectivity: An example from the Tertiary of the Faeroe–Shetland Basin, United Kingdom." *AAPG Bulletin* 88, no. 1 (2004): 1-20.

De Ruig, M. J., J. Smit, T. Geel, and H. Kooi. "Effects of the Pyrenean collision on the Paleocene stratigraphic evolution of the southern Iberian margin (southeast Spain)." *GSA Bulletin* 103, no. 11 (1991): 1504-1512.

den Hartog Jager, D., M. R. Giles, and G. R. Griffiths. "Evolution of Paleogene submarine fans of the North Sea in space and time." (1993) in J. R. Parker, ed., Petroleum geology of northwest Europe: London, The Geological Society, v. 1, pp. 59–71.

Deptuck, Mark E., R. Andrew MacRae, John W. Shimeld, Graham L. Williams, and Robert A. Fensome. "Revised Upper Cretaceous and lower Paleogene lithostratigraphy and depositional history of the Jeanne d'Arc Basin, offshore Newfoundland, Canada." *AAPG Bulletin* 87, no. 9 (2003): 1459-1483.

Dickinson, William R., Raymond V. Ingersoll, and Stephan A. Graham. "Paleogene sediment dispersal and paleotectonics in northern California." *GSA Bulletin* 90, no. 10 pt. II (1979): 1458-1528.

Dickinson, William R., Margaret A. Klute, Michael J. Hayes, Susanne U. Janecke, Erik R. Lundin, Mary A. McKittrick, and Mark D. Olivares. "Paleogeographic and paleotectonic setting of Laramide sedimentary basins in the central Rocky Mountain region." *GSA Bulletin* 100, no. 7 (1988): 1023-1039.

Dolan, James, Paul Mann, Ruurdjan de Zoeten, Christoph Heubeck, James Shiroma, and Simonetta Monechi. "Sedimentologic, stratigraphic, and tectonic synthesis of Eocene-Miocene sedimentary basins, Hispaniola and Puerto Rico." (1991) in Paul Mann, James Shiroma, and Simonetta Monechi, eds., Geologic and tectonic development of the North America-Caribbean Plate boundary in Hispaniola, GSA Special Papers 262: pp. 217-263.

Dumitru, Trevor A., W. G. Ernst, James E. Wright, Joseph L. Wooden, Ray E. Wells, Lucia P. Farmer, Adam JR Kent, and Stephan A. Graham. "Eocene extension in Idaho generated

massive sediment floods into the Franciscan trench and into the Tyee, Great Valley, and Green River basins." *Geology* 41, no. 2 (2013): 187-190.

Dziadzio, Piotr S., Mark A. Enfield, Matthew P. Watkinson, and Szczepan J. Porbski. "The Ciezkowice Sandstone: Examples of basin-floor fan-stacking patterns from the main (upper Paleocene to Eocene) reservoir in the Polish Carpathians." (2006) in J. Golonka and F. J. Picha, eds., The Carpathians and their foreland: Geology and hydrocarbon resources: AAPG Memoir 84: pp. 477-496.

Escalona, Alejandro, and Paul Mann. "Sequence-stratigraphic analysis of Eocene clastic foreland basin deposits in central Lake Maracaibo using high-resolution well correlation and 3-D seismic data." *AAPG Bulletin* 90, no. 4 (2006): 581-623.

Exon, N. F., Kennett, J. P., and Malone, M. J. The Cenozoic Southern Ocean: Tectonics, Sedimentation and Climate Change Between Australia and Antarctica, Geophysical Monograph Series, 151, American Geophysical Union, Washington, 367 pp. 2004.

Faupl, Peter, and Michael Wagreich. "Late Jurassic to Eocene palaeogeography and geodynamic evolution of the Eastern Alps." *Mitteilungen der Österreichischen Geologischen Gesellschaft* 92, no. 1999 (2000): 79-94.

Galloway, William E., Patricia E. Ganey-Curry, Xiang Li, and Richard T. Buffler. "Cenozoic depositional history of the Gulf of Mexico basin." *AAPG Bulletin* 84, no. 11 (2000): 1743-1774.

Galloway, W.E. “Gulf of Mexico Basin depositional record of Cenozoic North American drainage basin evolution.” (2005) in M. D. Blum, S. B. Marriott, S. F. Leclair, eds., Fluvial

Sedimentology VII: Special Publication of the International Association of Sedimentologists 35, pp. 409-423

Galloway, William E., Timothy L. Whiteaker, and Patricia Ganey-Curry. "History of Cenozoic North American drainage basin evolution, sediment yield, and accumulation in the Gulf of Mexico basin." *Geosphere* 7, no. 4 (2011): 938-973.

Gawenda, Piotr, Wilfried Winkler, Birger Schmitz, and Thierry Adatte. "Climate and bioproductivity control on carbonate turbidite sedimentation (Paleocene to earliest Eocene, Gulf of Biscay, Zumaia, Spain)." *Journal of Sedimentary Research* 69, no. 6 (1999): 1253-1261.

Görür, N., F. Y. Oktay, I. Seymen, and A. M. C. Şengör. "Palaeotectonic evolution of the Tuzgölü basin complex, Central Turkey: sedimentary record of a Neo-Tethyan closure." *Geological Society, London, Special Publications* 17, no. 1 (1984): 467-482.

Görür, Naci, Okan Tüysüz, and A. M. Celal Şengör. "Tectonic evolution of the central Anatolian basins." *International Geology Review* 40, no. 9 (1998): 831-850.

Graham, S. A., and Berry, K. D. “Early Eocene paleogeography of the central San Joaquin Valley: Origin of the Cantua Sandstone.” (1979) in J. M. Armentrout, M. R. Cole, and H. Ter Best Jr., eds., Cenozoic Paleogeography of the Western United States: Los Angeles, Pacific Section, SEPM (Society for Sedimentary Geology), Pacific Coast Paleogeography Symposium 3, pp. 119-127.

Grundvåg, Sten-Andreas, Erik P. Johannessen, William Helland-Hansen, and Piret Plink-Björklund. "Depositional architecture and evolution of progradationally stacked lobe

complexes in the Eocene Central Basin of Spitsbergen." *Sedimentology* 61, no. 2 (2014): 535-569.

Guardado, L. R., L. A. P. Gamboa, and C. F. Lucchesi. "Petroleum Geology of the Campos Basin, Brazil, a Model for a Producing Atlantic Type Basin." (1989) in J. D. Edwards and P. A. Santogrossi, eds., Divergent/passive margins: AAPG Memoir, v. 48, pp. 3-79.

Heritier, F. E., P. Lossel, and E. Wathne. "Frigg field—large submarine-fan trap in lower Eocene rocks of North Sea Viking graben." *AAPG Bulletin* 63, no. 11 (1979): 1999-2020.

Hesse, Reinhard. "Turbiditic and non-turbiditic mudstone of Cretaceous flysch sections of the East Alps and other basins." *Sedimentology* 22, no. 3 (1975): 387-416.

Karner, Garry D., and Neal W. Driscoll. "Tectonic and stratigraphic development of the West African and eastern Brazilian Margins: insights from quantitative basin modelling." *Geological Society, London, Special Publications* 153, no. 1 (1999): 11-40.

Knutsen, Stig-Morten, and Tore O. Vorren. "Early Cenozoic sedimentation in the Hammerfest Basin." *Marine Geology* 101, no. 1-4 (1991): 31-48.

Knutsen, Stig-Morten, Lars-Johan Skjold, and Pål Hermann Skott. "Palaeocene and Eocene development of the Tromsø Basin sedimentary response to rifting and early sea-floor spreading in the Barents Sea area." *Norsk geologisk tidsskrift* 72 (1992): 191-207.

Koop, W. J., and R. Stoneley. “Subsidence history of the Middle East Zagros basin, Permian to recent.” *Philosophical Transactions of the Royal Society of London. Series A, Mathematical and Physical Sciences* 305, no. 1489 (1982): 149-168.

Kumpulainen, Risto A. "Stratigraphy and sedimentology in western Nicaragua." *Revista Geologica de America Central* 18 (1995).

Labaume, P., M. Séguret, and C. Seyve. "Evolution of a turbiditic foreland basin and analogy with an accretionary prism: Example of the Eocene south-Pyrenean basin." *Tectonics* 4, no. 7 (1985): 661-685.

Lamers, E., and S. M. M. Carmichael. "The Paleocene deepwater sandstone play West of Shetland." In *Geological Society, London, Petroleum Geology Conference series*, vol. 5, no. 1, pp. 645-659. Geological Society of London, 1999.

Link, Martin H., and Tor H. Nilsen. "The Rocks Sandstone, an Eocene sand-rich deep-sea fan deposit, northern Santa Lucia Range, California." *Journal of Sedimentary Research* 50, no. 2 (1980): 583-601.

Lugo, Jairo, and Paul Mann. "Jurassic-Eocene tectonic evolution of Maracaibo basin, Venezuela." (1995) in A. J. Tankard, R. Suárez S., and H. J. Welsink, eds., Petroleum basins of South America: AAPG Memoir 62, pp. 699-725.

McGovney, J. E., and B. J. Radovich. "Seismic Stratigraphy and Facies of the Frigg Fan Complex: Chapter 8." (1985) in O. R. Berg and D. G. Woolverton, eds., Seismic stratigraphy II, an integrated approach: AAPG Memoir 39, pp. 139-156.

Menéndez, Leidy, Reinaldo Rojas-Consuegra, Jorge Villegas-Martín, and Rafael A. López. "Taphonomy, cronostratigraphy and paleoceanographic implications at turbidite of Early Paleogene (Vertientes Formation), Cuba." *Revista Geológica de América Central* 45 (2011): 87-94.

Modica, Christopher J., and Eugene R. Brush. "Postrift sequence stratigraphy, paleogeography, and fill history of the deep-water Santos Basin, offshore southeast Brazil." *AAPG Bulletin* 88, no. 7 (2004): 923-945.

Mohriak, W. U., M. R. Mello, J. F. Dewey, and J. R. Maxwell. "Petroleum geology of the Campos Basin, offshore Brazil." *Geological Society, London, Special Publications* 50, no. 1 (1990): 119-141.

Moore, J. G., and P. M. Shannon. "Palaeocene-Eocene deltaic sedimentation, Porcupine Basin, offshore Ireland-a sequence stratigraphic approach." *First Break* 10, no. 12 (1992): 461-469.

Morton, Andrew C. "Lower Tertiary sand development in Viking Graben, North Sea." *AAPG Bulletin* 66, no. 10 (1982): 1542-1559.

Moss, Steve J. "Embaluh Group turbidites in Kalimantan: evolution of a remnant oceanic basin in Borneo during the Late Cretaceous to Palaeogene." *Journal of the Geological Society* 155, no. 3 (1998): 509-524.

Mutti, Emiliano. "Distinctive thin-bedded turbidite facies and related depositional environments in the Eocene Hecho Group (South-central Pyrenees, Spain)." *Sedimentology* 24, no. 1 (1977): 107-131.

Mutti, Emiliano. "The Hecho Eocene submarine fan system, south-central Pyrenees, Spain." *Geo-Marine Letters* 3, no. 2-4 (1983): 199-202.

Nemčok, M., S. Rybár, P. Ekkertová, J. Kotulová, S. A. Hermeston, and D. Jones. "Transform-margin model of hydrocarbon migration: the Guyana–Suriname case study." *Geological Society, London, Special Publications* 431, no. 1 (2016): 199-217.

Nilsen, Tor H., Thomas W. Dibblee Jr, and Tully R. Simoni Jr. "Stratigraphy and sedimentology of the Cantua Sandstone Member of the Lodo Formation, Vallecitos area, California." (1974) in M. Payne, M., ed., *The Paleogene of the Panoche Creek–Cantua Creek Area, Central California:* Los Angeles, Pacific Section, SEPM (Society for Sedimentary Geology), Fall Field Trip Guidebook, pp. 38-68.

Nilsen, Tor H., and Tully R. Simoni Jr. "Deep-sea fan paleocurrent patterns of the Eocene Butano sandstone, Santa Cruz mountains, California." *U.S. Geological Survey Journal of Research* 1 (1973): 439-452.

Nilsen, Tor H., and Samuel H. Clarke. *Sedimentation and tectonics in the early Tertiary continental borderland of central California*. U.S. Geological Survey Professional Paper 925, 64 p. 1975.

Nilsen, Tor H. "Butano turbidite system, California." (1985) in A. H. Bouma, W. R. Normark, and N. E. Barnes, eds., *Submarine Fans and Related Turbidite Systems*, pp. 173-178. Springer, New York, NY.

Nyagah, Kivuti. "Stratigraphy, depositional history and environments of deposition of Cretaceous through Tertiary strata in the Lamu Basin, southeast Kenya and implications for reservoirs for hydrocarbon exploration." *Sedimentary Geology* 96, no. 1-2 (1995): 43-71.

Olivero, Eduardo Bernardo, and Norberto Malumián. "Mesozoic-Cenozoic stratigraphy of the Fuegian Andes, Argentina." *Geologica Acta* 6, no. 1 (2008): 0005-18.

Palermo, Denis, Mauro Galbiati, Massimo Famiglietti, Marco Marchesini, Domenico Mezzapesa, and Franco Fonnesu. "Insights into a new super-giant gas field-sedimentology and reservoir modeling of the Coral Reservoir Complex, Offshore Northern Mozambique." In *Offshore Technology Conference-Asia*. Offshore Technology Conference, 2014.

Palermo, D., M. Galbiati, D. Mezzapesa, M. Marchesini, F. Maioli, and F. Fonnesu. "Sequence Stratigraphy, Sedimentology and Reservoir Modelling of the Coral Reservoir, Offshore Northern Mozambique." In *Offshore Mediterranean Conference and Exhibition*. Offshore Mediterranean Conference, 2015.

Payros, Aitor, Xabier Orue-Etxebarria, and Victoriano Pujalte. "Covarying sedimentary and biotic fluctuations in Lower–Middle Eocene Pyrenean deep-sea deposits: palaeoenvironmental implications." *Palaeogeography, Palaeoclimatology, Palaeoecology* 234, no. 2-4 (2006): 258-276.

Payros, Aitor, and Naroa Martínez-Braceras. "Orbital forcing in turbidite accumulation during the Eocene greenhouse interval." *Sedimentology* 61, no. 5 (2014): 1411-1432.

Pickering, Kevin T., and Nicole J. Bayliss. "Deconvolving tectono-climatic signals in deep-marine siliciclastics, Eocene Ainsa basin, Spanish Pyrenees: Seesaw tectonics versus eustasy." *Geology* 37, no. 3 (2009): 203-206.

Plafker, George. "Regional geology and petroleum potential of the northern Gulf of Alaska continental margin." (1987) in D. W. Scholl, ed., Geology and Resource Potential of the Continental Margin of Western North America and Adjacent Ocean Basins: Houston, pp. 229-268.

Pudsey, C. J., and H. G. Reading. "Sedimentology and structure of the Scotland Group, Barbados." *Geological Society, London, Special Publications* 10, no. 1 (1982): 291-308.

Ramanathan, R., and E. Garcia. "Cretaceous paleogeography, foraminiferal biostratigraphy and palaeoecology of Belize Basin, Belize." 2nd Geological Conference of the Geological Society of Trinidad and Tobago (1991): 203-211.

Ranero, César R., Roland Huene, Ernst Flueh, Manuel Duarte, Dania Baca, and Kirk McIntosh. "A cross section of the convergent Pacific margin of Nicaragua." *Tectonics* 19, no. 2 (2000): 335-357.

Redwine, Lowell E. "The Tertiary Princeton submarine valley system beneath the Sacramento Valley, California." Ph.D. dissertation, University of California, Los Angeles, 1972, 480 p.

Reid, Stephen A. "Late Cretaceous and Paleogene sedimentation along the east side of the San Joaquin Basin." (1988) in S. A. Graham, ed., *Studies of the Geology of the San Joaquin Basin*: Pacific Section, Society of Economic Paleontologists and Mineralogists, v. 60, pp. 157-171.

Said, Aymen, Christoph Moder, Stuart Clark, and Badr Ghorbal. "Cretaceous–Cenozoic sedimentary budgets of the Southern Mozambique Basin: Implications for uplift history of the South African Plateau." *Journal of African Earth Sciences* 109 (2015): 1-10.

Salman, G., and I. Abdula. "Development of the Mozambique and Ruvuma sedimentary basins, offshore Mozambique." *Sedimentary Geology* 96, no. 1-2 (1995): 7-41.

Sansom, Pamela. "Hybrid turbidite–contourite systems of the Tanzanian margin." *Petroleum Geoscience* 24, no. 3 (2018): 258-276.

Santra, Manasij, Ronald J. Steel, Cornel Olariu, and Michael L. Sweet. "Stages of sedimentary prism development on a convergent margin—Eocene Tyee Forearc Basin, Coast Range, Oregon, USA." *Global and Planetary Change* 103 (2013): 207-231.

Schafhauser, A., W. Stinnesbeck, B. Holland, T. Adatte, and J. Remane. "Lower Cretaceous pelagic limestones in southern Belize: proto-Caribbean deposits on the southeastern Maya block." (2003) in C. Bartolini, R. T. Buffler, and J. Blickwede, eds., The Circum-Gulf of Mexico and the Caribbean: Hydrocarbon habitats, basin formation, and plate tectonics: AAPG Memoir 79, pp. 624-637.

Sekretov, Sergey B. "Northwestern margin of the East Siberian Sea, Russian Arctic: seismic stratigraphy, structure of the sedimentary cover and some remarks on the tectonic history." *Tectonophysics* 339, no. 3-4 (2001): 353-371.

Shannon, Patrick M. "Early Tertiary submarine fan deposits in the Porcupine Basin, offshore Ireland." *Geological Society, London, Special Publications* 62, no. 1 (1992): 351-373.

Sharman, Glenn R., Jacob A. Covault, Daniel F. Stockli, Anton F-J. Wroblewski, and Meredith A. Bush. "Early Cenozoic drainage reorganization of the United States Western Interior–Gulf of Mexico sediment routing system." *Geology* 45, no. 2 (2017): 187-190.

Siyako, M., and O. Huvaz. "Eocene stratigraphic evolution of the Thrace Basin, Turkey." *Sedimentary Geology* 198, no. 1-2 (2007): 75-91.

Sørensen, Aage Bach. "Cenozoic basin development and stratigraphy of the Faroes area." *Petroleum Geoscience* 9, no. 3 (2003): 189-207.

Soto, Matías, Ethel Morales, Gerardo Veroslavsky, Héctor de Santa Ana, Nelson Ucha, and Pablo Rodríguez. "The continental margin of Uruguay: Crustal architecture and segmentation." *Marine and Petroleum Geology* 28, no. 9 (2011): 1676-1689.

Speed, R. C. "Geology of Barbados: Implications for an accretionary origin." *Oceanologica Acta, Special issue* (1981).

Speed, R. C., and D. K. Larue. "Barbados: Architecture and implications for accretion." *Journal of Geophysical Research: Solid Earth* 87, no. B5 (1982): 3633-3643.

Steel, Ron, John Gjelberg, William Helland-Hansen, Karen Kleinspehn, Arvid Nøttvedt, and Morten Rye-Larsen. "The Tertiary strike-slip basins and orogenic belt of Spitsbergen." *SEPM Special Publication* 37 (1985): 339-360.

Steuer, Stephan, Dieter Franke, Florian Meresse, Dimitri Savva, Manuel Pubellier, Jean-Luc Auxietre, and Mario Aurelio. "Time constraints on the evolution of southern Palawan Island, Philippines from onshore and offshore correlation of Miocene limestones." *Journal of Asian Earth Sciences* 76 (2013): 412-427.

Struss, I., C. Brandes, M. Blisniuk, and J. Winsemann. "Eocene deep-water channel–levee deposits, Nicaragua: channel geometries and internal deformation patterns of six outcrops." Atlas of Deep-Water Outcrops: AAPG Studies in Geology 56 (2007).

Struss, I., V. Artiles, B. Cramer, and Jutta Winsemann. "The petroleum system in the Sandino forearc basin, offshore western Nicaragua." *Journal of Petroleum Geology* 31, no. 3 (2008): 221-244.

Suzuki, Shigeyuki, Shizuo Takemura, Graciano P. Yumul, Sevillo D. David, and Daniel K. Asiedu. "Composition and provenance of the Upper Cretaceous to Eocene sandstones in Central Palawan, Philippines: Constraints on the tectonic development of Palawan." *Island Arc* 9, no. 4 (2000): 611-626.

Swain, Frederick M. "Bottom sediments of lake Nicaragua and lake Managua, Western Nicaragua." *Journal of Sedimentary Research* 36, no. 2 (1966): 522-540.

Tailleur, Irvin L. "Probable rift origin of Canada basin, Arctic Ocean." *AAPG Bulletin* 54, no. 12 (1970): 2508-2508.

Taira, A., H. Okada, J. H. Whitaker, and A. J. Smith. "The Shimanto Belt of Japan: cretaceous-lower Miocene active-margin sedimentation." *Geological Society, London, Special Publications* 10, no. 1 (1982): 5-26.

Talukdar, Suhas, Oswaldo Gallango, and Marcel Chin-A-Lien. "Generation and migration of hydrocarbons in the Maracaibo Basin, Venezuela: An integrated basin study." *Organic Geochemistry* 10, no. 1-3 (1986): 261-279.

Torres Carbonell, Pablo J., and Eduardo B. Olivero. "Sand dispersal in the southeastern Austral Basin, Tierra del Fuego, Argentina: Outcrop insights from Eocene channeled turbidite systems." *Journal of South American Earth Sciences* 33, no. 1 (2012): 80-101.

Trümpy, Rudolf. "Paleotectonic evolution of the Central and Western Alps." *GSA Bulletin* 71, no. 6 (1960): 843-907.

Ujiie, Kohtaro. "Evolution and kinematics of an ancient décollement zone, mélange in the Shimanto accretionary complex of Okinawa Island, Ryukyu Arc." *Journal of Structural Geology* 24, no. 5 (2002): 937-952.

Underwood, Michael B. "Depositional setting of the Paleogene Yager Formation, northern Coast Ranges of California." (1983) in D. K. Larue, and R. J. Steel, eds., Cenozoic marine sedimentation, Pacific margin, U.S.A.: Los Angeles, Pacific Section, Society of Economic Paleontologists and Mineralogists, pp. 81-101.

Villegas-Martín, Jorge, Renata Guimaraes Netto, Ernesto Luis Correa Lavina, and Reinaldo Rojas-Consuegra. "Ichnofabrics of the Capdevila Formation (early Eocene) in the Los Palacios Basin (western Cuba): Paleoenvironmental and paleoecological implications." *Journal of South American Earth Sciences* 56 (2014): 214-227.

Walford, H. L., N. J. White, and J. C. Sydow. "Solid sediment load history of the Zambezi Delta." *Earth and Planetary Science Letters* 238, no. 1-2 (2005): 49-63.

Willumsen, P. S., and R. P. Cote. "Tertiary sedimentation in the southern Beaufort Sea, Canada." (1982) in A. F. Embry and H. R. Balkwill, eds., Arctic Geology and Geophysics: Proceedings of the Third International Symposium on Arctic Geology: Canadian Society of Petroleum Geologists Memoir 8, pp. 43-53.

Winker, Charles D. "Cenozoic shelf margins, northwestern Gulf of Mexico." *Gulf Coast Association of Geological Societies Transactions* 32 (1982): 427-448.

Winkler, Gary R. “Deep-sea fan deposition of the lower Tertiary Orca Group, eastern Prince William Sound, Alaska.” *U.S. Geological Survey Open-File Report*, no. 76-83 (1976): 20 p.

Xie, Xiangyang, Paul Mann, and Alejandro Escalona. "Regional provenance study of Eocene clastic sedimentary rocks within the South America–Caribbean plate boundary zone using detrital zircon geochronology." *Earth and Planetary Science Letters* 291, no. 1-4 (2010): 159-171.

Zhang, Jinyu, Jacob Covault, Michael Pyrcz, Glenn Sharman, Cristian Carvajal, and Kristy Milliken. "Quantifying sediment supply to continental margins: Application to the Paleogene Wilcox Group, Gulf of Mexico." *AAPG Bulletin* 102, no. 9 (2018): 1685-1702.

Zongying, Zhou, Tao Ye, Li Shujun, and Ding Wenlong. "Hydrocarbon potential in the key basins in the East Coast of Africa." *Petroleum Exploration and Development* 40, no. 5 (2013): 582-591.

References: Table S2

Abbott, Lon D., Eli A. Silver, Peter R. Thompson, Mark V. Filewicz, and Cindy Schneider. "Stratigraphic constraints on the development and timing of arc-continent collision in northern Papua New Guinea." *Journal of Sedimentary Research* 64, no. 2b (1994): 169-183.

Alava, Jorge Toro, and Etienne Jaillard. "Provenance of the Upper Cretaceous to upper Eocene clastic sediments of the Western Cordillera of Ecuador: Geodynamic implications." *Tectonophysics* 399.1-4 (2005): 279-292.

Alsharhan, A. S., and A. E. M. Nairn. "Tertiary of the Arabian Gulf: sedimentology and hydrocarbon potential." *Palaeogeography, Palaeoclimatology, Palaeoecology* 114, no. 2-4 (1995): 369-384.

Armentrout, John M., and David H. Suek. "Hydrocarbon exploration in western Oregon and Washington." *AAPG Bulletin* 69, no. 4 (1985): 627-643.

Bader, Richard G., et al. Initial Reports of the Deep Sea Drilling Project, Volume IV. (1970) Washington (U.S. Government Printing Office).

Baillie, P. W., C. M. Powell, Z. X. Li, and A. M. Ryall. "The tectonic framework of Western Australia's Neoproterozoic to Recent sedimentary basins." (1994) in P. G. Purcell, and R. R. Purcell, eds., *West Australian Basins Symposium*: Proceedings of the Petroleum Exploration Society of Australia, Perth, pp. 45-62.

Balkwill, Hugh R. "Evolution of Sverdrup basin, arctic Canada." *AAPG Bulletin* 62, no. 6 (1978): 1004-1028.

Bastia, Ravi. "An overview of Indian sedimentary basins with special focus on emerging east coast deepwater frontiers." *The Leading Edge* 25, no. 7 (2006): 818-829.

Beavington-Penney, Simon J., V. Paul Wright, and Andrew Racey. "The middle Eocene Seeb Formation of Oman: an investigation of acyclicity, stratigraphic completeness, and accumulation rates in shallow marine carbonate settings." *Journal of Sedimentary Research* 76, no. 10 (2006): 1137-1161.

Beh, Richard L. "Evolution and geology of western Baffin Bay and Davis Strait, Canada." (1975) in C. J. Yorath, E. R. Parker, and D. J. Glass, eds., Canada's Continental Margins and Offshore Petroleum Exploration: Canadian Society of Petroleum Geology Memoirs, 4, pp. 453-476.

Biswas, S. K. "Rift basins in western margin of India and their hydrocarbon prospects with special reference to Kutch basin." *AAPG Bulletin* 66, no. 10 (1982): 1497-1513.

Bourillet, J.-F., S. Zaragosi, and T. Mulder. "The French Atlantic margin and deep-sea submarine systems." *Geo-Marine Letters* 26, no. 6 (2006): 311-315.

Bowland, Christopher L. "Depositional history of the western Colombian Basin, Caribbean Sea, revealed by seismic stratigraphy." *GSA Bulletin* 105, no. 10 (1993): 1321-1345.

Brannan, J., K. D. Gerdes, and I. R. Newth. "Tectono-stratigraphic development of the Qamar basin, Eastern Yemen." *Marine and Petroleum Geology* 14, no. 6 (1997): 701-730.

Brownfield, Michael E., and Ronald R. Charpentier. *Assessment of the undiscovered oil and gas of the Senegal Province, Mauritania, Senegal, the Gambia, and Guinea-Bissau, Northwest Africa*. Vol. 25. Denver: US Department of the Interior, US Geological Survey, 2003.

Bueno Salazar, Rafael, and Carlos Govea. "Potential for exploration and development of Hydrocarbons in Atrato Valley and Pacific Coastal and Shelf Basins of Colombia." *AAPG Bulletin* 25 (1974): 318-327.

Bueno Salazar, Rafael. "Hydrocarbon exploration and potential of the Pacific coastal basin of Colombia." (1989) in G. E. Ericksen, M. T. Canas Pinochet, and J. A. Reinemund, eds., Geology of the Andes and its relation to hydrocarbon and mineral resources: Houston, Texas, Circum-Pacific Council for Energy and Mineral Resources Earth Science Series, v. 11.

Bushnell, David C., Jorge E. Baldi, Fernando H. Bettini, Humberto Franzin, Edward Ned Kovas, Raul Marinelli, and Grant J. Wartenburg. "Petroleum systems analysis of the eastern Colorado Basin, offshore northern Argentina." (2000) in M. R. Mello and B. J. Katz, eds., Petroleum systems of South Atlantic margins: AAPG Memoir 73, pp. 403–415.

Bustin, R. M. "Organic maturation and petroleum source rock potential of Tofino Basin, southwestern British Columbia." *Bulletin of Canadian Petroleum Geology* 43, no. 2 (1995): 177-186.

Butcher, B. P. “Northwest Shelf of Australia.” (1990) in J. D. Edwards and P. A. Santogrossi, eds., Divergent/Passive Margin Basins: AAPG Memoir 48, pp. 81-115.

Carter, Andrew, Yani Najman, Abbas Bahroudi, Paul Bown, Eduardo Garzanti, and Robert D. Lawrence. "Locating earliest records of orogenesis in western Himalaya: Evidence from Paleogene sediments in the Iranian Makran region and Pakistan Katawaz basin." *Geology* 38, no. 9 (2010): 807-810.

Charusiri, Punya, and Somchai Pum-Im. "Cenozoic tectonic evolution of major sedimentary basins in central, northern, and the Gulf of Thailand." *Bull. Earth Sci. Thail.* 2 (2009): 40-62.

Chen, Percy PH, Zhi Yuong Chen, and Qi Min Zhang. "Sequence stratigraphy and continental margin development of the northwestern shelf of the South China Sea." *AAPG Bulletin* 77, no. 5 (1993): 842-862.

Cooper, M. A., F. T. Addison, R. Alvarez, M. Coral, R. H. Graham, A. B. Hayward, S. Howe et al. "Basin development and tectonic history of the Llanos Basin, Eastern Cordillera, and middle Magdalena Valley, Colombia." *AAPG Bulletin* 79, no. 10 (1995): 1421-1442.

Damuth, John E. "Neogene gravity tectonics and depositional processes on the deep Niger Delta continental margin." *Marine and Petroleum Geology* 11, no. 3 (1994): 320-346.

Davies, Peter J., Philip A. Symonds, David A. Feary, and Christopher J. Pigram. "The evolution of the carbonate platforms of northeast Australia." *SEPM Special Publication* 44 (1989): 233-258.

Di Croce, Juan. "Eastern Venezuela Basin: Sequence stratigraphy and structural evolution." Ph.D. dissertation, Rice University, 1996.

Dunbar, Robert B., Richard C. Marty, and Paul A. Baker. "Cenozoic marine sedimentation in the Sechura and Pisco basins, Peru." *Palaeogeography, Palaeoclimatology, Palaeoecology* 77, no. 3-4 (1990): 235-261.

Edgar, N. Terence, Saunders, John B., et al. Initial Reports of the Deep Sea Drilling Project, Volume 15. (1973) Washington (U.S. Government Printing Office).

Eldrett, James S., Ian C. Harding, John V. Firth, and Andrew P. Roberts. "Magnetostratigraphic calibration of Eocene–Oligocene dinoflagellate cyst biostratigraphy from the Norwegian–Greenland Sea." *Marine Geology* 204, no. 1 (2004): 91-127.

Ewing, John, Manik Talwani, Maurice Ewing, and Terence Edgar. "Sediments of the Caribbean." *Studies in Tropical Oceanography* 5 (1967): 88-102.

Feary, David A., Peter J. Davies, Christopher J. Pigram, and Philip A. Symonds. "Climatic evolution and control on carbonate deposition in northeast Australia." *Palaeogeography, Palaeoclimatology, Palaeoecology* 89, no. 4 (1991): 341-361.

Fildani, Andrea, Angela M. Hessler, and Stephan A. Graham. "Trench-forearc interactions reflected in the sedimentary fill of Talara basin, northwest Peru." *Basin Research* 20, no. 3 (2008): 305-331.

Fisher, Michael A., William W. Patton Jr, and Mark L. Holmes. "Geology of Norton Basin and continental shelf beneath northwestern Bering Sea, Alaska." *AAPG Bulletin* 66, no. 3 (1982): 255-285.

Franke, Dieter, Soenke Neben, Bernd Schreckenberger, Albrecht Schulze, Manfred Stiller, and Charlotte M. Krawczyk. "Crustal structure across the Colorado Basin, offshore Argentina." *Geophysical Journal International* 165, no. 3 (2006): 850-864.

Gardosh, Michael A., and Yehezkel Druckman. "Seismic stratigraphy, structure and tectonic evolution of the Levantine Basin, offshore Israel." *Geological Society, London, Special Publications* 260, no. 1 (2006): 201-227.

Garzanti, Eduardo, Pieter Vermeesch, Sergio Andò, Giovanni Vezzoli, Manuel Valagussa, Kate Allen, Khalid A. Kadi, and Ali IA Al-Juboury. "Provenance and recycling of Arabian desert sand." *Earth-Science Reviews* 120 (2013): 1-19.

Gradstein, F. M., and S. P. Srivastava. "Aspects of Cenozoic stratigraphy and paleoceanography of the Labrador Sea and Baffin Bay." *Palaeogeography, Palaeoclimatology, Palaeoecology* 30 (1980): 261-295.

Grant, A. C., K. D. McAlpine, and J. A. Wade. "The continental margin of eastern Canada: geological framework and petroleum potential." (1986) in M.T. Halbouty, ed., Future Petroleum Provinces of the World: AAPG Memoir 40, pp. 177-205.

Gruetzner, Jens, Gabriele Uenzelmann-Neben, and Dieter Franke. "Variations in sediment transport at the central Argentine continental margin during the Cenozoic." *Geochemistry, Geophysics, Geosystems* 13, no. 10 (2012).

Hamilton, Edwin L. "Marine geology of abyssal plains in the Gulf of Alaska." *Journal of Geophysical Research* 72, no. 16 (1967): 4189-4213.

Henriksen, E., A. E. Ryseth, G. B. Larssen, T. Heide, K. Rønning, K. Sollid, and A. V. Stoupakova. "Tectonostratigraphy of the greater Barents Sea: implications for petroleum systems." *Geological Society, London, Memoirs* 35, no. 1 (2011): 163-195.

Hou, B., N. F. Alley, L. A. Frakes, L. Stoian, and W. M. Cowley. "Eocene stratigraphic succession in the Eucla Basin of South Australia and correlation to major regional sea-level events." *Sedimentary Geology* 183, no. 3-4 (2006): 297-319.

Huang, Chi-Yue, Yi Yen, QuanHong Zhao, and Chiou-Ting Lin. "Cenozoic stratigraphy of Taiwan: Window into rifting, stratigraphy and paleoceanography of South China Sea." *Chinese Science Bulletin* 57, no. 24 (2012): 3130-3149.

Iturralde-Vinent, Manuel A., Consuelo Díaz Otero, Antonio García-Casco, and Douwe J. J. van Hinsbergen. "Paleogene foredeep basin deposits of north-central Cuba: A record of arc-continent collision between the Caribbean and North American plates." *International Geology Review* 50, no. 10 (2008): 863-884.

Jaillard, Etienne, Martha Ordoñez, Stalin Benitez, Gerardo Berrones, Nelson Jiménez, Galo Montenegro, and Italo Zambrano. "Basin development in an accretionary, oceanic-floored fore-arc setting: southern coastal Ecuador during late Cretaceous-late Eocene time." (1995) in A. J. Tankard, R. Suarez Soruco, and H. J. Welsink, eds., Petroleum Basins of South America: AAPG Memoir 62, pp. 597-613.

Jaillard, E., G. Hérail, T. Monfret, E. Díaz-Martínez, P. Baby, A. Lavenu, and J. F. Dumont. "Tectonic evolution of the Andes of Ecuador, Peru, Bolivia and northernmost Chile." (2000) in U. G. Cordani, E. J. Milani, A. Thomaz Filho, and D. A. Campos, eds., Tectonic Evolution of South America: 31st International Geological Congress, Rio de Janeiro, pp. 481–559.

Kamp, P. J. J., K. A. Vincent, M. J. S. Tayler, 2015. Cenozoic sedimentary and volcanic rocks of New Zealand: A reference volume of lithology, age and paleoenvironments with maps (PMAPs) and database. Ministry of Business, Innovation and Employment, New Zealand, unpublished Petroleum Report PR4885, 335 pp.

Larsen, Michael, Christian Knudsen, Dirk Frei, Martina Frei, Thomas Rasmussen, and Andrew G. Whitham. "East Greenland and Faroe–Shetland sediment provenance and Palaeogene sand dispersal systems." *Geological Survey of Denmark and Greenland Bulletin* 10 (2006): 29-32.

Lee, Gwang H., and Joel S. Watkins. "Seismic sequence stratigraphy and hydrocarbon potential of the Phu Khanh Basin, offshore central Vietnam, South China Sea." *AAPG Bulletin* 82, no. 9 (1998): 1711-1735.

Lee, Gwang H., Keumsuk Lee, and Joel S. Watkins. "Geologic evolution of the Cuu Long and Nam Con Son basins, offshore southern Vietnam, South China Sea." *AAPG Bulletin* 85, no. 6 (2001): 1055-1082.

Lin, A. T., Anthony Brian Watts, and S. P. Hesselbo. "Cenozoic stratigraphy and subsidence history of the South China Sea margin in the Taiwan region." *Basin Research* 15, no. 4 (2003): 453-478.

Macgregor, Duncan S. "Late Cretaceous–Cenozoic sediment and turbidite reservoir supply to South Atlantic margins." *Geological Society, London, Special Publications* 369 (2012): 109-128.

Marcaillou, Boris, and Jean-Yves Collot. "Chronostratigraphy and tectonic deformation of the North Ecuadorian–South Colombian offshore Manglares forearc basin." *Marine Geology* 255, no. 1-2 (2008): 30-44.

Marsaglia, K. M., and A. V. Carozzi. "Depositional environment, sand provenance, and diagenesis of the Basal Salina Formation (lower Eocene), northwestern Peru." *Journal of South American Earth Sciences* 3, no. 4 (1990): 253-267.

Maurizot, Pierre. "First sedimentary record of the pre-obduction convergence in New Caledonia: formation of an Early Eocene accretionary complex in the north of Grande Terre and emplacement of the 'Montagnes Blanches' nappe." *Bulletin de la Société Géologique de France* 182, no. 6 (2011): 479-491.

Maurizot, Pierre, and Dominique Cluzel. "Pre-obduction records of Eocene foreland basins in central New Caledonia: an appraisal from surface geology and Cadart-1 borehole data." *New Zealand Journal of Geology and Geophysics* 57, no. 3 (2014): 300-311.

McIver, N. L. "Cenozoic and Mesozoic stratigraphy of the Nova Scotia shelf." *Canadian Journal of Earth Sciences* 9, no. 1 (1972): 54-70.

McWhae, J. R. H., R. Elie, K. C. Laughton, and P. R. Gunther. "Stratigraphy and petroleum prospects of the Labrador Shelf." *Bulletin of Canadian Petroleum Geology* 28, no. 4 (1980): 460-488.

Mills, Richard A., and K. E. Hugh. "Reconnaissance geologic map of Mosquitia region, Honduras and Nicaragua Caribbean coast." *AAPG Bulletin* 58, no. 2 (1974): 189-207.

Mills, Richard A., and R. Barton. "Geology of the Ahuas area in the Mosquitia basin of Honduras: Preliminary report." *AAPG Bulletin* 80, no. 10 (1996): 1627-1640.

Monnier, Damien, P. Imbert, Aurélien Gay, R. Mourgues, and Michel Lopez. "Pliocene sand injectites from a submarine lobe fringe during hydrocarbon migration and salt diapirism: a seismic example from the Lower Congo Basin." *Geofluids* 14, no. 1 (2014): 1-19.

Morris, John C. "The stratigraphy of the Amuri limestone group, east Marlborough, New Zealand." Ph.D. dissertation, University of Canterbury, 1987, 388 p.

Nygren, W. E. "Bolivar geosyncline of northwestern South America." *AAPG Bulletin* 34, no. 10 (1950): 1998-2006.

Paton, Douglas A., David van der Spuy, Rolando di Primio, and Brian Horsfield. "Tectonically induced adjustment of passive-margin accommodation space; influence on the hydrocarbon potential of the Orange Basin, South Africa." *AAPG Bulletin* 92, no. 5 (2008): 589-609.

Poag, C. Wylie, and William D. Sevon. "A record of Appalachian denudation in postrift Mesozoic and Cenozoic sedimentary deposits of the US middle Atlantic continental margin." *Geomorphology* 2, no. 1-3 (1989): 119-157.

Polachan, Songpope, Surawit Pradidtan, Chalermkiat Tongtaow, Somkiat Janmaha, Kanok Intarawijitr, and Chutamat Sangsuwan. "Development of Cenozoic basins in Thailand." *Marine and Petroleum Geology* 8, no. 1 (1991): 84-97.

Qayyum, Mazhar, Alan R. Niem, and Robert D. Lawrence. "Newly discovered Paleogene deltaic sequence in Katawaz basin, Pakistan, and its tectonic implications." *Geology* 24, no. 9 (1996): 835-838.

Qayyum, Mazhar, Robert D. Lawrence, and Alan R. Niem. "Discovery of the palaeo-Indus delta-fan complex." *Journal of the Geological Society* 154, no. 5 (1997a): 753-756.

Qayyum, Mazhar, Robert D. Lawrence, and Alan R. Niem. "Molasse-delta-flysch continuum of the Himalayan orogeny and closure of the Paleogene Katawaz remnant ocean, Pakistan." *International Geology Review* 39, no. 10 (1997b): 861-875.

Qayyum, Mazhar, Alan R. Niem, and Robert D. Lawrence. "Detrital modes and provenance of the Paleogene Khojak Formation in Pakistan: Implications for early Himalayan orogeny and unroofing." *GSA Bulletin* 113, no. 3 (2001): 320-332.

Quilty, Patrick G. "Cenozoic sedimentation cycles in Western Australia." *Geology* 5, no. 6 (1977): 336-340.

Ren, Jianye, Kensaku Tamaki, Sitian Li, and Zhang Junxia. "Late Mesozoic and Cenozoic rifting and its dynamic setting in Eastern China and adjacent areas." *Tectonophysics* 344, no. 3-4 (2002): 175-205.

Rohr, K. M. M., and J. R. Dietrich. "Strike-slip tectonics and development of the Tertiary Queen Charlotte Basin, offshore western Canada: Evidence from seismic reflection data." *Basin Research* 4, no. 1 (1992): 1-20.

Ryseth, Alf, Jan Harald Augustson, Mike Charnock, Ole Haugerud, Stig-Morten Knutsen, Peter S. Midbøe, Jan Gunnar Opsal, and Gyrd Sundsbø. "Cenozoic stratigraphy and evolution of the Sørvestsnaget Basin, southwestern Barents Sea." *Norwegian Journal of Geology/Norsk Geologisk Forening* 83, no. 2 (2003): 107-130.

Safronova, Polina A., Sverre Henriksen, Karin Andreassen, Jan Sverre Laberg, and Tore O. Vorren. "Evolution of shelf-margin clinoforms and deep-water fans during the middle Eocene in the Sørvestsnaget Basin, southwest Barents Sea." *AAPG Bulletin* 98, no. 3 (2014): 515-544.

Sastri, V. V., R. N. Sinha, Gurcharan Singh, and K. V. S. Murti. "Stratigraphy and tectonics of sedimentary basins on east coast of peninsular India." *AAPG Bulletin* 57, no. 4 (1973): 655-678.

Schenk, Christopher J. "Geology and petroleum potential of the West Greenland–East Canada Province." *Geological Society, London, Memoirs* 35, no. 1 (2011): 627-645.

Scholl, David W., Roland von Huene, and James B. Ridlon. "Spreading of the ocean floor: undeformed sediments in the Peru-Chile Trench." *Science* 159, no. 3817 (1968): 869-871.

Scholl, David W., Mark N. Christensen, Roland von Huene, and Michael S. Marlow. "Peru-Chile trench sediments and sea-floor spreading." *GSA Bulletin* 81, no. 5 (1970): 1339-1360.

Scholl, David W., Edwin C. Buffington, and Michael S. Marlow. "Plate tectonics and the structural evolution of the Aleutian–Bering Sea region." In *GSA Special Papers*, pp. 1-31. The Geological Society of America, 1975.

Seranne, Michel, and César-Rostand Nzé Abeigne. "Oligocene to Holocene sediment drifts and bottom currents on the slope of Gabon continental margin (west Africa): Consequences for sedimentation and southeast Atlantic upwelling." *Sedimentary Geology* 128, no. 3-4 (1999): 179-199.

Snavely Jr, Parke D. "Tertiary geologic framework, neotectonics, and petroleum potential of the Oregon-Washington continental margin." (1987) in D. W. Scholl, A. Grantz, and J.G. Vedder, eds., Geology and resource potential of the continental margin of western North America and adjacent ocean basins— Beaufort Sea to Baja California: Circum-Pacific Council for Energy and Mineral Resources, Earth Sci. Series, v. 6, pp. 305-335.

Snavely Jr, Parke D., and Ray E. Wells. "Cenozoic evolution of the continental margin of Oregon and Washington." (1996) in A.M. Rogers et al., eds., *Assessing Earthquake Hazards and Reducing Risk in the Pacific Northwest*, U.S. Geol. Surv. Prof. Pap., 1560, pp. 161-182.

Stevenson, Andrew J., and Robert Embley. "Deep-sea fan bodies, terrigenous turbidite sedimentation, and petroleum geology, Gulf of Alaska." (1987) in D. W. Scholl, A. Grantz, and J. G. Vedder, eds., Geology and Resource Potential of the Continental Margin of Western North America and Adjacent Ocean Basins – Beaufort Sea to Baja California: Circum-Pacific Council for Energy and Mineral Resources, Earth Science Series, vol. 6, p. 503-522.

Swezey, Christopher S. "Cenozoic stratigraphy of the Sahara, northern Africa." *Journal of African Earth Sciences* 53, no. 3 (2009): 89-121.

Thornton, Scott E., Gerald Kidd, Terry Stellman, Hector del Castillo, Peter Mullin, Edwin Goter, and Pratt Barndollar. "Straddling the Basin Boundary in Equatorial Guinea and Gabon: Contrasting Structural Style, Trapping Styles and Reservoirs." *Houston Geological Society Bulletin* 56, no. 02 (2013): 25, 27, 29, 31.

Tiffin, D. L., B. E. B. Cameron, and J. W. Murray. "Tectonics and depositional history of the continental margin off Vancouver Island, British Columbia." *Canadian Journal of Earth Sciences* 9, no. 3 (1972): 280-296.

Toelsie, Sharmila, and Prediepkoemar Goerdajal. "Sand control in shallow unconsolidated sandstone oil reservoirs at Staatsolie NV Suriname." In *SPE European Formation Damage Conference & Exhibition*. Society of Petroleum Engineers, 2013.

Travis, R. B., Gonzales, G. and Pardo, A., 1976. “Hydrocarbon potential of coastal basins of Peru.” (1976) in M. Halbouty et al., eds., Circum-Pacific Energy Resources: AAPG Memoir 25, pp. 331-338.

Tuttle, Michele L., Ronald R. Charpentier, and Michael E. Brownfield. *The Niger Delta Petroleum System: Niger Delta Province, Nigeria, Cameroon, and Equatorial Guinea, Africa*. U.S. Department of the Interior, U.S. Geological Survey, 1999.
